# Overexpression of *Meloe* Gene in Melanomas Is Controlled Both by Specific Transcription Factors and Hypomethylation

**DOI:** 10.1371/journal.pone.0075421

**Published:** 2013-09-25

**Authors:** Mathilde Bobinet, Virginie Vignard, Laetitia Florenceau, Francois Lang, Nathalie Labarriere, Agnès Moreau-Aubry

**Affiliations:** 1 Institut National de la Santé et de la Recherche Médicale, UMR 892, Nantes, France; 2 University of Nantes, Nantes, France; 3 Centre national de la recherche scientifique, UMR 6299, Nantes, France; 4 University hospital of Nantes, Nantes, France; University of Tennessee, United States of America

## Abstract

The melanoma antigens MELOE-1 and MELOE-2 are encoded by a messenger, called *meloe*, overexpressed in melanomas compared with other tumour cell types and healthy tissues. They are both able to elicit melanoma-specific T cell responses in melanoma patients, and MELOE-1-specific CD8 T cells have been involved in melanoma immunosurveillance. With the aim to develop immunotherapies targeting this antigen, we investigated the transcriptional mechanisms leading to the preferential expression of *meloe* messenger in the melanocytic lineage. We defined the minimal promoter region of *meloe* gene and identified binding motifs for a set of transcription factors. Using mutagenesis, co-transfection experiments and chromatin immunoprecipitation, we showed that transcription factors involved in *meloe* promoter activity in melanomas were the melanocytic specific SOX9 and SOX10 proteins together with the activated P-CREB protein. Furthermore, we showed that *meloe* promoter was hypomethylated in melanomas and melanocytes, and hypermethylated in colon cancer cell lines and mesotheliomas, thus explaining the absence of P-CREB binding in these cell lines. This was a second key to explain the overerexpression of *meloe* messenger in the melanocytic lineage. To our knowledge, such a dual transcriptional control conferring tissue-specificity has never been described for the expression of tumour antigens.

## Introduction

We previously identified a new transcript *meloe* (melanoma overexpressed antigen) over-expressed in human melanomas and under-expressed in other tumour cell types or healthy tissues. This transcript appears specifically expressed in humans, since *in silico* analyses did not reveal any homologous transcript in other mammalian species. This unconventional mRNA codes for at least two antigens namely MELOE-1 and MELOE-2 recognized by melanoma specific T cells in the HLA-A2 context [1], [2]. We also showed that the adjuvant infusion of MELOE-1 specific CD8 T cells prolonged relapse-free survival of melanoma patients treated by adoptive transfer of tumour infiltrating lymphocytes [1]. The immunological interest of MELOE-1 protein was further strengthened by the discovery of a vast CD8 T cell repertoire specific for this antigen, in all HLA-A2 patients [3] and by the characterization of multiple class II helper epitopes from this antigen [4], [5]. These properties make this antigen an attractive target for immunotherapy protocols of melanoma, and thus we sought to investigate the transcriptional mechanisms leading to the overexpression of *meloe* in the melanocytic lineage.

Aside from mutated antigens, tumour antigens can be divided into different groups based on their expression profile in healthy and malignant tissues. Classical overexpressed antigens, such as P53 [6] and Telomerase [7], are highly expressed in a variety of tumour cells and at a lower level in their normal cell counterparts. Genes coding cancer germline antigens (such as MAGE genes), are in turn expressed in many different tumours but are silent in normal cells, except in male germline cells [8]. Finally, tissue differentiation antigens, such as melanocytic antigens [9], are specifically expressed in a cell lineage, including tumour and healthy tissues. None of these expression profiles corresponds exactly to that of *meloe* messenger, overexpressed in melanomas but weakly expressed in other tumour cell types and healthy tissues [1], suggesting a regulation specific of melanomas.

Two main mechanisms have been involved in the regulation of tumour antigen expression: regulation by tissue specific transcription factors (TFs), responsible for the expression of differentiation antigens and hypomethylation of the gene promoters in tumour tissues, as reported for cancer germline antigens, such as MAGE, BAGE, GAGE and NY-ESO-1 antigens [10], [11], [12]. Such epigenetic alteration in the cancer cell genome leads to a shared expression of these tumour antigens between different types of cancers, that does not fit with *meloe* expression profile. Differentiation antigens, in turn, are exclusively expressed in the melanocytic lineage, such as Melan-A, gp100, Tyrosinase or TYRP1. Their tissue specificity is conferred by melanocytic specific TFs, such as micropthalmia associated TF (MITF) [13], [14], [15], [16]. Unlike these antigens, *meloe* expression was not formally restricted to the melanocytic lineage as a residual expression can be detected in other cancer cell types, however, at a level too low to induce the activation of MELOE-1 or MELOE-2 specific T lymphocytes [1], [2]. Thus, in this study we investigated the mechanisms responsible for the unusual expression profile of *meloe* messenger, first by defining its minimal functional promoter and then by looking for TFs and regulation mechanisms involved in its overexpression in melanomas.

## Materials and Methods

### Tumor cell lines

Melanoma cell lines were established in the GMP Unit of Cellular Therapy and in our laboratory from lymph node metastases and belong to the Biocollection PCU892-NL (CHU Nantes). Written consents were obtained from all patients. This biocollection was approved by the local ethic committee of Nantes hospital (GNDES), and registered under the CNIL number «1278197». Human Mesothelioma cell lines, Meso45, Meso61, Meso85 and Meso163, previously characterized [17] and belonging to the Biocollection PCU892-MG were gifts from M. Grégoire (INSERM U892, Nantes, France). Breast cancer cell line MCF-7, lung carcinoma cell lines A549 and H69, colorectal carcinoma cell lines HCT116, SW707 and SW480, renal carcinoma cell line A498 and neuroblastoma cell line SH-SY5Y were obtained from the ATCC. Melanocytes were gifts from M. Regnier (L'Oréal Laboratory, Paris, France). Cell lines were cultured in RPMI-1640 medium, or DMEM medium for the neuroblastoma cell line, supplemented with 10% heat-inactivated fetal calf serum (PAA), 2 nM L-glutamine, 100 IU/mL penicillin and 0.1 mg/mL streptomycin (Gibco).

### qPCR

RNA samples, extracted from tumour cell lines and melanocytes by RNA purification system NucleoSpin RNA II (Macherey-Nagel, Hoerdt-FRANCE) exhibited an RNA integrity number >9. Retrotranscription was performed using 1 μg of total RNA, oligodT, and SuperScript III reverse transcriptase (Invitrogen-Life-Technologies, Saint-Aubin-FRANCE). Relative quantification of *meloe* and house keeping genes (HKG) RPLPO and cyclophilin-A expression was performed using Brilliant SYBR Green qPCR (Stratagene-Agilent Technologies, Les-Ulis-FRANCE). cDNA samples (20 ng) were added to SYBR Green Master Mix with specific primers at 200 nM. *RPLPO*: 5′-GTGATGTGCAGCTGATCAAGACT-3′ and 5′-GATGACCAGCCCAAAGGAGA-3′; *cyclophilin-A*: 5′-CCACCGTGTTCTTCGACAT-3′ and 5′-CCAGTGCTCAGAGCACGAAA-3′; *meloe*: 5′-GTCCTCCCCAGCACCAGAGT-3′ and 5′-AGCCTGCCATCTGCAATCCT-3′. For the three genes, thermal cycling was 95°C for 10′, 40 cycles at 95°C for 30′′, 60°C for 1′, and 72°C for 1′. The efficiency of PCR reaction was validated with duplicate series of 10-fold-diluted cDNA from the melanoma cell line M113, performed in parallel to plot the standard curves for the three genes. Mean threshold cycle (CT) values from duplicate PCR reactions were normalized to mean CT values for the two HKG from the same cDNA preparations. The relative expression ratio of a target gene was calculated based on the PCR efficiency (E) and the CT deviation between a given cell line (x) and a calibrator (eight melanocyte cultures), expressed in comparison with the mean of the HKG: ratio  =  (E target)^ ΔCT target (calibrator – x)^/mean ((E HKG) ^ΔCT HKG (calibrator – x)^).

### 5′RACE PCR

5′RACE PCR was performed using the SMART RACE cDNA amplification method [18], [19]. 2 µg of polyA RNA extracted from M113 melanoma cell line (Ambion-Life Technologies) were reversely transcribed using 12 µM of the following primers: 5′-CDS: 5′-(T)_25_VN-3′, and SMART primers: 5′- AAGCAGTGGTATCAACGCAGAGTACGC**r**G**r**G**r**G -3′ (Eurofins, Nantes-FRANCE), with 200U Superscript III reverse transcriptase (Life technologies). The first strain synthesized was used for 5′-RACE PCR, using an UPM primer mix (2 µM UPM-long/10 µM UPM-short) (5′-ctaatacgactcactatagggcAAGCAGTGGTATCAACGCAGAGT-3′ and 5′-ctaatacgactcactatagggc-3′) and a gene specific primer: 5′-ccagcttctccagcagtttagcg-3′. PCR amplification was carried out as follows: 98°C, 30′′; 35 cycles 98°C, 10′′; 70°C, 45′′; 72°C, 45′′. A nested PCR was performed using the Nup primer: 5′-aagcagtggtatcaacgcagagt-3′ and a gene-specific primer: 5′-gaagggatgttcacactgccttg-3′ under the same conditions as the first PCR.

The PCR product was ligated into the pSC-B-amp/kan cloning vector (Stratagene-Agilent Technologies). The cloned 5′RACE constructs were sequenced by the DNA Sequencing Facility of the SFR Sante.

### Construction of truncated and mutated promoters

All constructs using *Firefly* luciferase reporter gene were performed in the promoterless pGL4.10 vector (Promega, Charbonnières-FRANCE). Various truncated *meloe* promoters were generated by PCR with a shared reverse primer: 5′-ccatggtggcgaagggatgttcacactgccttgg-3′. Forward primers were designed with restriction sites for subcloning in pGL4.10, at various positions starting from −1565 bp. The promoter fragment of the *melan-A* gene, spanning 230 nucleotides, was used as a positive control [20].

Mutated promoters were generated using the QuikChange™ Site-Directed Mutagenesis Kit (Stratagene) with the following modifications: MITF [−659] catgtg→TTtTtT, MITF [+495] cacgtg→cacCtC, PAX3 [−633] ggtgacgttt→gCCCCcgttt, ETS [−407] agaggaa→agaCCGa, Activator Protein 1 (AP1) [−354] tgagtca→ACTgtca, Sry-box (SOX) [−93] ctttgt→ctttAT, CRE binding protein (CREB) [−80] ctgacgtca→ctgaTgtca.

### Plasmid transfections and Dual Luciferase Reporter assay

Cells were transfected with a mixture of 300ng of the pGL4.10 *Firefly* luciferase vector under *meloe* promoter control and 20 ng of *Renilla* luciferase vector pRL-CMV (Promega), with Lipofectamine (0.5 µL, Invitrogen) in serum free medium. For co-transfection experiments, 50 to 150 ng of co-transfected or empty vector were added to the transfection mixture. After 2 h30, complete medium was added to the transfection mixture. After 48 h, luciferase activities of cell extracts were measured using the Dual-Glo Luciferase assay system (Promega). For each experiment, the average ratio of the *Firefly* luciferase to the *Renilla* luciferase activity of three independent transfections was calculated.

SOX9 cDNA was purchased from Open Biosystems (Thermo-scientific). SOX10 wild type and mutant cDNA (E189X) were a gift from M. Goossens and N. Bondurand (IMRB, Créteil-FRANCE) [21].

### Western blotting analysis

Tumour cell lines were lysed with RIPA lysis buffer supplemented with 5 mM NAF and protease inhibitors (Roche, Boulogne-Billancourt-FRANCE). Lysates were sonicated and centrifuged. 10–50 ug of total proteins were separated on 8% SDS-polyacrylamide electrophoresis gels and blotted onto PVDF membranes (Millipore, Molsheim-FRANCE). Membranes were incubated with diluted primary antibodies against SOX9 (AB5535, Millipore), SOX10 (sc-17342, Santa-Cruz Biotechnology-Inc, Heidelberg-GERMANY) or P-CREB (87G3, Cell-Signalling, Danvers, MA-USA), overnight at 4°C. Membranes were then incubated during 1h with appropriate HRP-conjugated secondary antibody, followed by ECL detection. Blots were stripped with stripping buffer (Pierce, Rockford, IL-USA) and reprobed for actin (MAB1501, Millipore) as control.

### Chromatin Immuno-precipitation (ChIP)

Chromatin was purified from 2.10^7^ tumour cells after cross-linking with 1% formaldehyde for 10′ at room temperature and then DNA was sonicated. ChIP assays were performed with the ChIP-IT™ kit (ActiveMotif, La Hulpe-BELGIUM) with the following antibodies: anti-SOX9 (Millipore) and anti-SOX10 (Santa-Cruz), anti-phospho-CREB (Cell-Signalling). To ensure result's reliability, two control samples have been included: the input sample and the chromatin precipitated with an irrelevant control antibody (anti-GFP, Roche), indicative of the amount of background signal. PCR amplification (from −137 to +89 bp), was performed on 1/10 of precipitated DNA and 1/100 of input DNA: 95°C, 5′; 36 cycles of 95°C, 30′′; 66°C, 1′, 72°C, 30′′, with the following primers: 5′-ATTCACAGCACACTGACCGTCT-3′ and 5′-TTGACCCAAAGCACCCTGAAGA-3′.

### DNA extraction and methylation status analyses

DNA from tumour cells was extracted by using the QiaAmp DNA mini Kit (Qiagen, Courtaboeuf-FRANCE). Human Melanocyte-adult genomic DNA was purchased from CliniSciences (Nanterre-FRANCE). Methylated DNA conversion was performed using the MethylCollector™ Bisulfite Modification Kit (Active Motif). DNA converted was amplified by two successive PCR with the following primers for PCR1: 5′-TGAGTTATTTTTTATTTGAAGAGATTTTAA-3′ and 5′-CACCTCCTACATTTTCACTCATTATAA-3′, PCR2: 5′-TTGATTGTGTTATTTAAAGAATAGTGTTTT-3′ and 5′-AAAATATTCACACTACCTTAATTTACC-3′. PCR cycles were: 95°C, 5′; then 20 cycles: 95°C, 30′′; 55°C, 2′; 72°C, 2′; final extension 72°C, 5′. The amplimer was inserted into pSC-B-Amp/Kan cloning vector (Stratagene). At least ten clones from each tissue were sequenced.

## Results

### Meloe is overexpressed in melanomas and melanocytes

**Figure 1:**
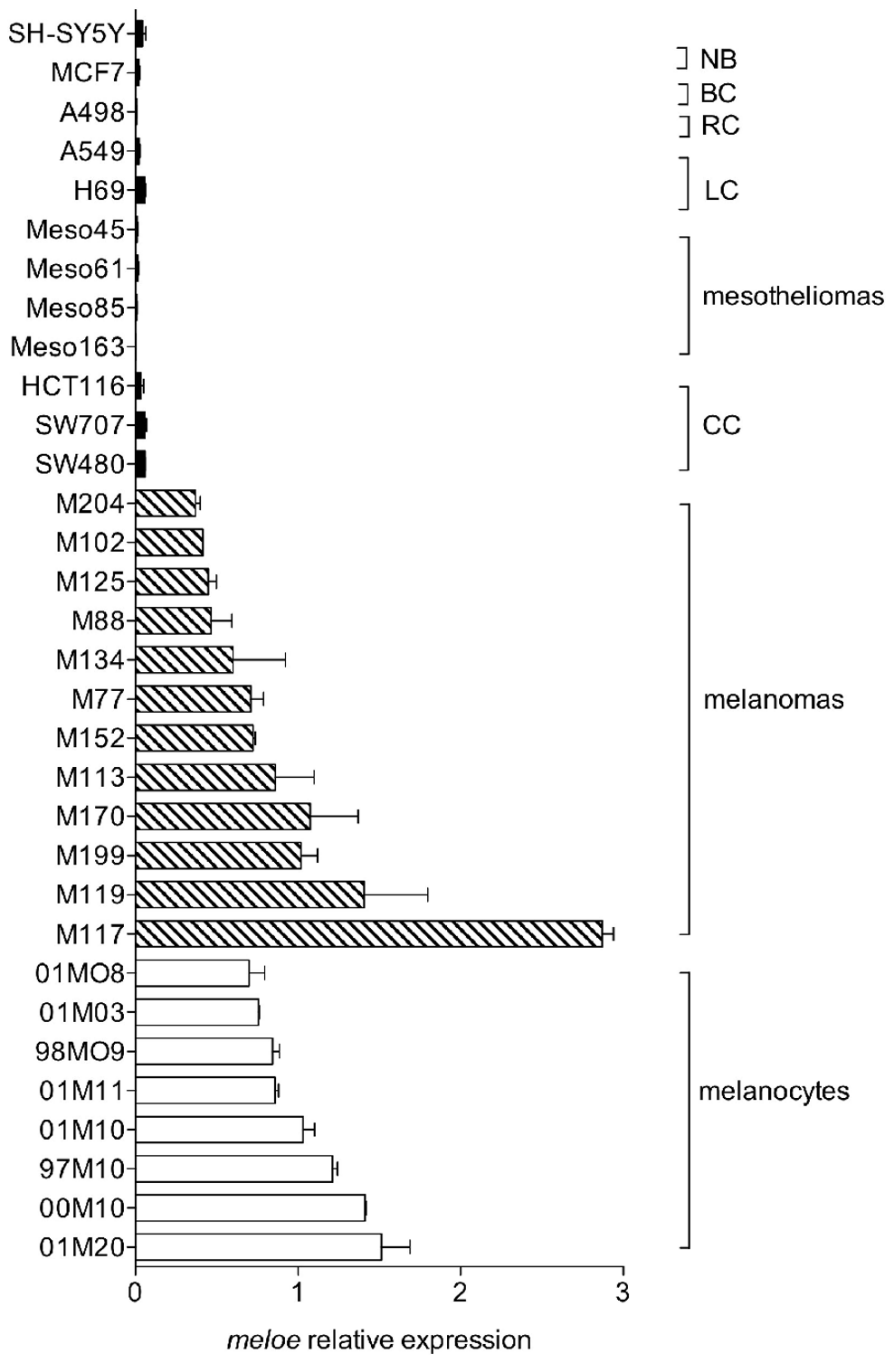
Overexpression of meloe cDNA in the melanocytic lineage. Eight melanocyte samples, twelve melanoma, three colon cancer (CC), four mesothelioma, two lung cancer (LC), one renal carcinoma (RC), one breast cancer (BC) and one neuroblastoma (NB) cell lines were tested by qPCR for the expression of *meloe*. RPLPO and cyclophilin-A gene expression were used as internal controls. The relative expression of *meloe* was calculated after normalization on the efficiency of PCR reaction and the mean expression of these two housekeeping genes, reported to its normalized expression in a mixture of eight distinct melanocyte samples. Results are from three independent experiments.

To strengthen our previous results concerning the expression profile of *meloe* in the melanocytic lineage compared to cancer cell lines of different origins [1], we expanded our sampling of melanocytes and melanoma cell lines, for extensive qPCR analysis. We also tested a number of house-keeping genes (HKG), and choose two genes whose expression remained constant between all the tested tumour cell lines and the melanocytes. As shown in Figure 1, the overexpression of *meloe* was confirmed in melanomas and more surprisingly in melanocytes, compared with other tumour cell types, such as colon, renal, lung, breast, mesotheliomas and neuroblastoma cell lines. In our previous study, we found that *meloe* was expressed to a lower extent in two of these same melanocytes (01MO8 and 98MO9) [1]. This discrepancy can be explained by the HKG used in our previous study. Indeed, one of them (ß2-microglobulin) was expressed at a higher level in melanocytes than in melanoma cell lines, thus artificially decreasing *meloe* relative expression. The very low expression of *meloe* in other healthy tissues was confirmed using the new HKG (data not shown), thus we can assert that this antigen is a tumour antigen overexpressed in the melanocytic lineage.

### Minimal promoter definition and validation of transcription factor binding sites

*meloe* messenger comprises multiple short ORFs (Open Reading Frame). The most proximal ORF shown to be translated is MELOE-2 [2]. We first defined the transcription initiation site by 5′ RACE PCR analysis starting from MELOE-2 proximal 5′ region. The longest amplified sequence places the start codon 544 bp upstream of MELOE-2 ATG, and thus presented 259 additional bases compared to the public sequence of *meloe* transcript [NR_026664].

**Figure 2:**
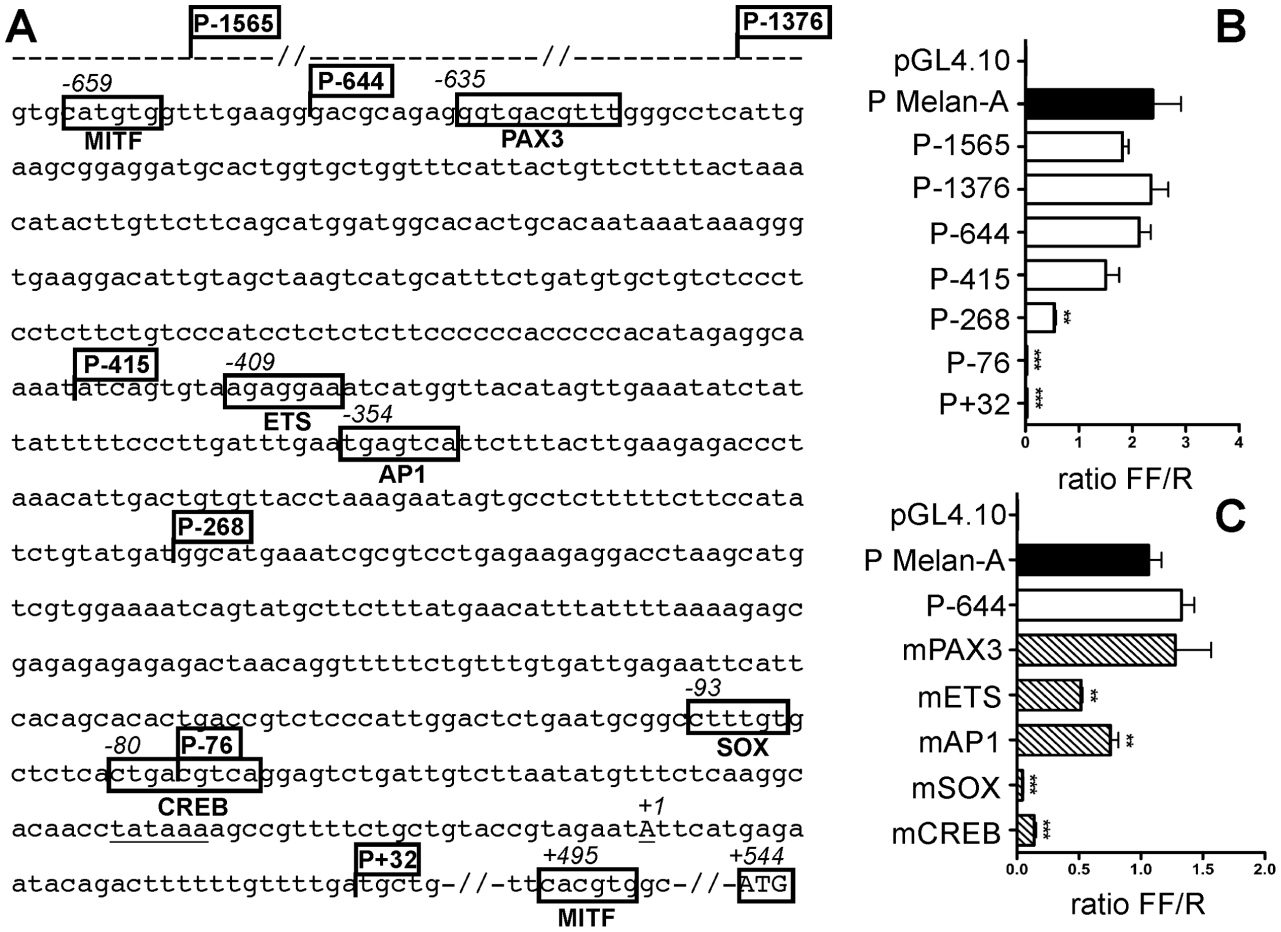
Minimal promoter definition and TF binding sites. (A) Sequence of the 5′ flanking region of *meloe*. Consensus sequences for TF are framed and flags illustrate positions of truncated promoters. The transcriptional start site and the putative TATAbox are underlined. (B) 5′ truncated or (C) mutated promoters were transfected into M113 melanoma cell line together with the *Renilla* luciferase pRL-CMV. The pGL4.10 empty vector was used as negative control and a Melan-A promoter as a positive one (black bar). Results, presented as *Firefly/Renilla* luciferase ratios (FF/R), are mean with SD from three independent experiments, performed in triplicate. Statistical analysis was performed using one-way ANOVA followed by a Dunnett's multiple comparison test, with P-1565 as reference for truncated promoters and P-644 for mutated ones (** p<0.01, ***p<0.001).

*In silico* analysis of *meloe* proximal promoter region (from −2000 to +1 nucleotide), located in the third intron of the HDAC-4 gene (NG_009235.1) [1], conducted with Jaspar database (Jaspar http://jaspar.cgb.ki.se/), highlighted the putative TATA element located 30bp upstream of the +1 transcription start and a number of motifs able to bind TFs, some of them illustrated on Figure 2A. We focused on TFs involved in melanocytic differentiation or melanoma progression. We found two E-boxes (CA [T/C]GTG) consensus sequences (−659 and +495) able to bind MITF [22], along with ETS (−409), AP1 (−354), SOX (−93), and CREB (−80) consensus sequences. We also identified a potential PAX3 consensus sequence (−635) sharing features with common patterns validated in a previous study [23].

In order to define the minimal promoter region and validate each of these consensus sequences, we conducted deletion and mutational approaches with a reporter plasmid, replacing MELOE-2 sequence by the *Firefly* luciferase gene. Each construct was transfected in the melanoma cell line M113, together with a control plasmid coding *Renilla* luciferase. The promoter of the melanocytic differentiation antigen Melan-A was used as a positive control [20]. Results first show that *meloe* promoter (from P-1565 to P-415) is as active as Melan-A promoter in melanoma cells (Figure 2B). Promoter activity decreased when the region between −415 and −268 (containing ETS and AP1 consensus sequences) was deleted (p<0.01), and was completely abrogated after deletion of the region containing SOX and CREB consensus sequences (P-76) (p<0.001). To formally validate these putative binding sites, we tested the activity of the promoter region P-644, containing mutated consensus sequences (Figure 2C). A significant decrease in luciferase activity was observed with promoters mutated on ETS or AP1 binding sites (p<0.01), in line with the decrease of promoter activity observed with P-268 truncated plasmid. Mutation of SOX and CREB binding sites entailed a drastic loss of promoter activity (P<0.001). Thus, SOX and CREB seem to be key factors involved in *meloe* transcription. As expected from deletion experiments, the mutation of the putative PAX3 binding site does not affect promoter activity. Finally, mutation of the two potential MITF binding sites (−659 and +495), performed on longer and shorter promoter regions (P-1376 and P-415), did not affect promoter activity (data not shown).

### Validation of the activator role of SOX9 and SOX10 on meloe promoter activity

**Figure 3:**
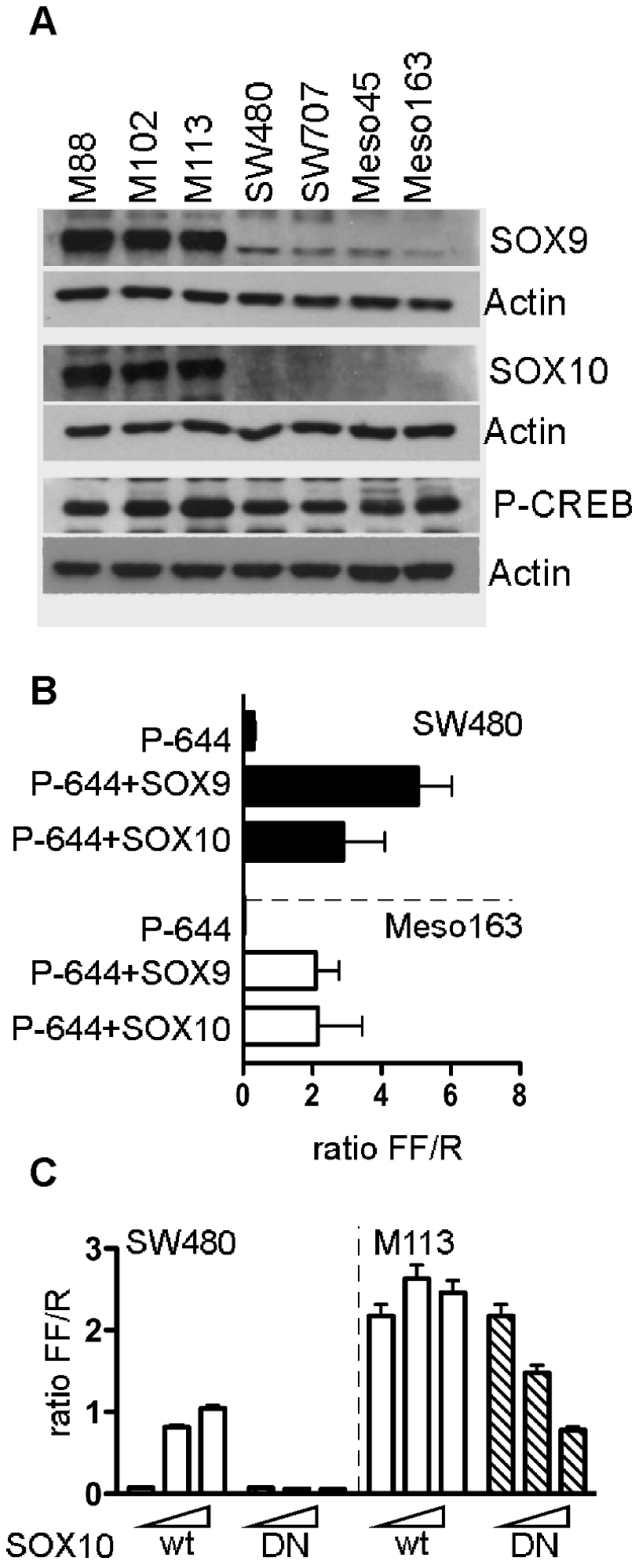
SOX9 and SOX10 are involved in meloe promoter activity. (A) Western-blot analysis of SOX9, SOX10 and P-CREB expression in melanoma, mesothelioma and colon carcinoma cell lines. (B) SW480 colon carcinoma and Meso163 mesothelioma cells were transfected with P-642 alone or with SOX9 or SOX10 expression plasmids together with the pRL-CMV. (C) SW480 and M113 cell lines were transfected with the P-644 vector alone or with a range (100 ng or 150 ng) of SOX10 expression plasmid (SOX10wt, empty bars) or its mutated form (SOX10DN, hatched bars), together with the pRL-CMV. Results, presented as *Firefly/Renilla* luciferase ratios (FF/R), are mean with SD from three (B) or two (C) independent experiments, performed in triplicate.

Among the SOX factor family, SOX9 and SOX10 belong to the SOX-E factors implicated in melanogenesis regulation. They are both expressed in melanocytes and melanomas and involved in the transcription of various genes [24], [25]. We assessed the expression of these two factors, together with the active P-CREB in melanoma cell lines, compared with two other tumour cell types that poorly or did not express *meloe* gene (colon carcinoma cell lines and mesotheliomas). As shown in Figure 3A, SOX9 is strongly expressed in melanoma cells, but weakly in colon carcinoma cells or mesotheliomas. SOX10, in turn, is expressed exclusively in melanoma cells. As expected, P-CREB is detected in each cell lysate.

In order to validate the implication of SOX9 and/or SOX10 factors in *meloe* promoter activation, we co-transfected expression vectors coding these factors, together with the P-644 plasmid in a colon carcinoma cell line (SW480) and a mesothelioma cell line (Meso163). The presence of each of these factors restores *meloe* promoter activity in these cell lines, strongly suggesting their involvement in *meloe* transcription in melanoma cells (Figure 3B).

A complementary approach was conducted using a dominant-negative SOX10 factor. This mutated form (E189X) contains the high-mobility-group binding domain but is defective for the activation of gene transcription [21]. The lack of activity of this truncated form, compared to the wild type form, was first confirmed upon co-transfection into SW480 cell line, with the P-644 *meloe* promoter plasmid (Figure 3C, left panel). Conversely, its co-transfection into M113 cell line with the P-644 plasmid strongly decreases promoter activity in a dose-dependent manner, by competing with endogenous SOX9 and SOX10 proteins. As expected, co-transfection of increasing doses of SOX10 wild type form has no effect in these cells spontaneously expressing high levels of SOX10 and SOX9 proteins (Figure 3C, right panel).

### Validation of the activator role of SOX9/SOX10 and P-CREB by ChIP

**Figure 4:**
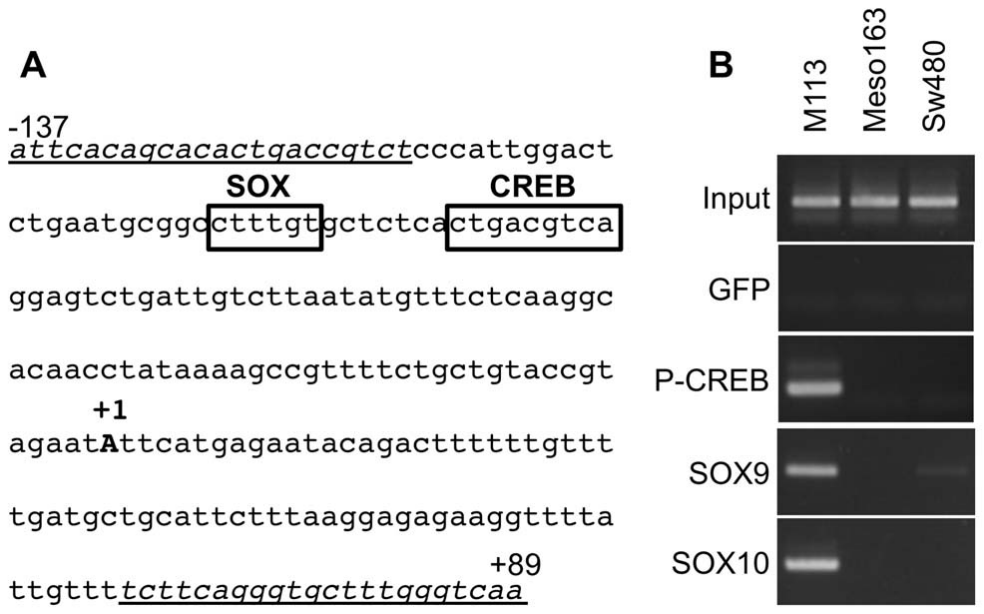
Validation of SOX9, SOX10 and CREB binding in vivo by ChIP. (A) *meloe* promoter region amplified on immunoprecipitated DNA. Forward and reverse primers used for PCR amplification are indicated in italics and underlined, and CREB and SOX binding sites are framed. (B) Chromatin Immunoprecipitation assay was performed on three cell lines: M113, Meso163 and SW480. Immunoprecipitations were performed with anti-P-CREB, anti-SOX9 and anti-SOX10 antibodies or with anti-GFP antibody as negative control. PCR amplification was performed on the immunoprecipitated DNA or on the input sample (positive control of non precipitated DNA) using primers spanning from −137 to +89.

To demonstrate that SOX9, SOX10 and P-CREB are actually recruited on *meloe* promoter *in vivo*, a ChIP experiment focusing on a restricted promoter region bearing SOX and CREB binding sites (Figure 4A) was conducted on three tumour cell lines: M113 cell line strongly expressing *meloe*, and two non melanoma cell lines poorly expressing *meloe* (Meso163 and SW480). The results show that these three factors are recruited on *meloe* promoter in melanoma cells. None of these factors is recruited on *meloe* promoter in mesothelioma cells, consistent with the very low expression of *meloe* in these cells. Finally, only SOX9 seemed to be fixed on *meloe* promoter in the colon carcinoma cell line, to a lesser extend compared with melanoma cells (Figure 4B).

### Meloe proximal promoter methylation status

**Figure 5:**
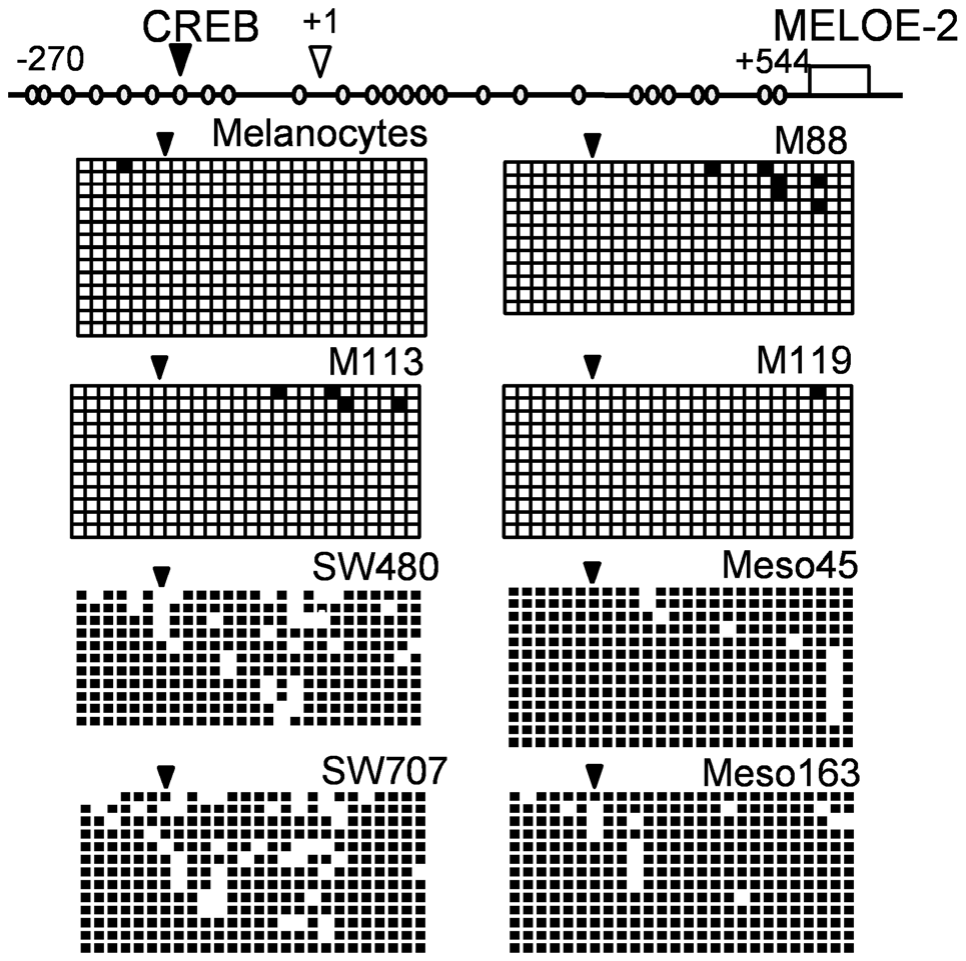
Methylation status of meloe region [−270–+544] in melanocytes, melanoma, mesothelioma and colon carcinoma cell lines. Each of the 26 CpG motifs included in the explored region was illustrated by empty circles (upper panel). The CpG island included in the CREB binding site is indicated by an arrow. DNA treated by bi-sulfite conversion was amplified, cloned and sequenced. Methylation status of this region in each cell line was represented by a grid where each line corresponds to one allele and each column to one CpG motif, methylated (black) or unmethylated (white).

In order to explain the absence of binding of P-CREB on *meloe* promoter in non-melanoma cell lines, we evaluated the methylation status of *meloe* proximal promoter. Indeed, it is widely documented that transcription efficiency depends on the methylation status of CpGs on promoter' genes, allowing or not the binding of TFs. The CREB binding site contains a CpG dinucleotide, whose methylation could impair CREB binding. Thus, a bisulfite conversion of genomic DNA from adult melanocytes, melanomas, mesotheliomas and colon carcinoma cell lines, was performed. Methylation status of the 26 CpGs included in the *meloe* region spanning from −270bp to +544 bp was analyzed (Figure 5). Results show that this region is almost totally unmethylated in melanocytes and melanoma cells. On the contrary, most of the CpG motifs of this region, including that of the CREB binding site, are hypermethylated in mesothelioma cells (100% in Meso45 and 77% in Meso163), and in colon carcinoma cells (64% in SW480 and 85% in SW707). The hypermethylation of the promoter region is consistent with the underexpression of *meloe* gene in non-melanocytic cells.

## Discussion

By qPCR analysis, we showed that *meloe* messenger was overexpressed only in the melanocytic lineage, although a weak expression can be found in other cancer cell types, such as colon carcinoma cell lines (Figure 1), and in healthy tissues ([1], and data not shown). This expression profile does not fit with that of a differentiation antigen, expressed in a specific lineage and totally absent in other tissues, nor with that of a cancer germline antigen normally expressed in germ cells and trophoblast tissues and aberrantly expressed in a variety of human malignancies [26]. In order to characterize mechanisms involved in *meloe* transcription, we first defined the minimal promoter region active in melanoma cells and look for TF binding sites that could be relevant in the melanocytic lineage. As shown on Figure 2C, CREB and SOX binding sites appeared essential for promoter activity whereas AP-1 and ETS binding sites seemed only involved in optimal promoter activation. Conversely, the putative PAX3 binding site (−635) did not seem involved in promoter activity, as well as the two proximal and distal putative MITF binding sites, that were actually E-boxes (CA [T/C]GTG) known to bind factors belonging to the basic helix-loop-helix (bHLH) and bHLH leucine zipper (bHLH-LZ) families. MITF, a bHLH-LZ TF critical for the regulation of melanocyte functions, recognizes an «AGTCA [T/C]GTG» DNA motif termed “M-box”, identified on gene promoters regulated by this factor, such as Tyrosinase or TRP-1 [27], [28]. It has to be stressed that the two E-boxes found on the analyzed sequence were not in the context of a M-box, thus not promoting MITF binding. In conclusion, these putative binding sites are not involved in promoter activity, but this does not exclude a possible fixation of PAX3 and/or MITF at more distant sites, and their possible role in *meloe* transcription regulation.

The AP1 family of bHLH-LZ TFs includes JUN and FOS that heterodimerize to form DNA-binding complexes and stimulate transcription of genes containing the AP1 consensus DNA-binding site TGA(C/G)TCA [29]. In melanomas, it has been demonstrated that c-JUN protein, together with CEBP/ß TF, was involved in transcriptional regulation of a specific melanoma differentiation associated gene (mda-7) [30]. The family of ETS factors regulate a number of genes implicated in tumour progression such as those coding metalloproteinases [31]. In melanomas, ETS-1 has been recently identified as a key factor in upregulation of Mcl-1 gene, upon endoplasmic reticulum stress, and thus in the resistance of melanoma cells to apoptosis [32]. Members of these two TF families could thus contribute to *meloe* overexpression in melanoma cells and melanocytes, without being absolutely necessary for minimal promoter activity.

Conversely, SOX and CREB proteins appeared essential for *meloe* expression as mutations of their specific binding sites completely abrogate promoter activity (Figure 2C). For this reason, we focused this first study on these factors, absolutely needed for *meloe* transcription. Furthermore, we also checked the absence of single nucleotide polymorphisms in these two binding sites, by *in silico* analysis [33], in order to exclude any inter-individual variation.

CREB is an ubiquitous bHLH-LZ TF that regulates the expression of numerous genes suppressing apoptosis, inducing cell proliferation, mediating inflammation and tumour metastases [34]. It has been involved in the tumorigenicity and metastatic potential of melanomas, through the regulation of expression of metalloproteinases and adhesion molecules [35], [36]. SOX TFs are grouped into nine classes. Among them, those participating in melanocyte differentiation and melanogenesis belong to the SOX-E group, including SOX9 and SOX10 [37], both expressed in established melanoma cell lines. SOX10 factor is crucial for the melanocytic development process, because of its regulation of MITF gene [38], but after the establishment of melanocyte precursor, SOX9 plays a similar role in this differentiation process in adults [39]. SOX9 and SOX10, overexpressed in melanomas [40], are implicated in the regulation of genes involved in melanogenesis such as MITF and tyrosinase [25].

We therefore endeavoured to formally demonstrate the implication of CREB, SOX9 and SOX10 factors in *meloe* promoter activity, first by evaluating their expression in melanoma cells. As expected, the activated form of CREB protein (P-CREB) was present in melanoma, colon cancer and mesothelioma cell lysates (Figure 3A). SOX9 protein was strongly present in melanoma cells, and weakly detected in colon cancer and mesothelioma cell lines whereas SOX10 expression was restricted to melanoma cell lines. These results are consistent with known SOX9 and SOX10 expression profiles [41]. The involvement of these two factors in *meloe* promoter activity has been further validated either upon co-transfection of wild-type SOX9 and SOX10 factors into non-melanoma cell lines (Figure 3B), or using a SOX10 dominant-negative form co-transfected into melanoma cells (Figure 3C). ChIP experiments further formally demonstrated the *in vivo* binding of SOX9, SOX10 and P-CREB on *meloe* promoter region in melanomas (Figure 4). Considering the proximity of SOX and CREB binding sites (13bp), we can hypothesise that they could act synergistically, as previously shown for MITF promoter [42].

Considering the ChiP experiments, none of these three factors (not even the ubiquitous CREB protein) was bound to *meloe* promoter in mesotheliomas, and only SOX9 was poorly bound to this region in colon carcinoma cells. Since the CREB binding is dependent of CpG dinucleotide demethylation included in the CRE motif, we further investigated the methylation status of proximal *meloe* promoter in these three tumour cell types and in melanocytes. The region from −270 to +544 bp of *meloe* was almost unmethylated in melanoma cell lines and in melanocytes, and hypermethylated in colon carcinoma cell lines and even more in mesotheliomas (Figure 5). The CpG dinucleotide of CREB binding site is thus hypermethylated in non-melanoma cell lines and likely impairs P-CREB binding in these cells, contributing to the under-expression of *meloe* gene. In cancer cells, epigenetic modifications are very frequent, regulating positively or negatively gene expression. Many tumours show hypomethylation of their genome, but the hypomethylation of the region of 800bp surrounding *meloe* transcription start site concerns both melanomas and their normal counterparts and thus appears specific to the melanocytic lineage. In the same line of thought, a melanoma specific hypomethylation has been described for the first intron of FOXP3 gene, demethylated in regulatory T cell clones and in melanoma cells, compared to other cancer cells such as lung and colorectal carcinoma cells [43].

To our knowledge, such a dual transcriptional control conferring tissue specificity for a gene coding tumor antigens has never been described, and the mechanisms leading to the melanoma specific hypomethylation of *meloe* gene will be explored in depth.
